# Chronic Alcohol Use and Accelerated Brain Aging: Shared Mechanisms with Alzheimer’s Disease Pathophysiology

**DOI:** 10.3390/brainsci16010035

**Published:** 2025-12-26

**Authors:** Nishtha Singh, Shouvik Kumar Nandy, Aditi Sharma, Vansh, Arif Jamal Siddiqui, Lalit Sharma

**Affiliations:** 1 Department of Pharmacology, School of Pharmaceutical Sciences, Shoolini University, Solan 173229, Himachal Pradesh, India; dogranishtha93@gmail.com (N.S.); shouvikknandy@gmail.com (S.K.N.); aditisharma31790@gmail.com (A.S.); vanshdogra40@gmail.com (V.); 2 Department of Biology, College of Science, University of Ha’il, Ha’il P.O. Box 2440, Saudi Arabia

**Keywords:** Alzheimer’s disease, alcohol consumption, neurodegeneration, amyloid-β, tau protein, oxidative stress, neuroinflammation, hippocampus, cognitive decline

## Abstract

Alzheimer’s disease (AD) is a progressive neurodegenerative disorder. Recent findings suggest that long-term and heavy alcohol consumption can aggravate several pathological processes associated with AD, whereas the impact of light or moderate consumption remains uncertain. Excessive alcohol exposure impairs the structure and function of key brain regions involved in cognition, particularly the hippocampus, prefrontal cortex, amygdala, cerebellum, Basolateral amygdala (BLA), and hypothalamus. Several studies indicate that chronic alcohol consumption affects the brain by multiple mechanisms like increased oxidative stress, microglial activation, neuroinflammation, microtubule instability, tau hyperphosphorylation, and modified amyloid-β turnover. Disruption of cholinergic transmission further contributes to memory deficits and neuronal susceptibility. These alcohol-related alterations closely resemble core features of AD pathology and may accelerate disease progression. Although some epidemiological studies report the potential benefits of low alcohol intake, their interpretation is limited by inconsistent definitions of drinking patterns and the influence of confounding variables. Overall, current evidence supports a dose-dependent relationship in which alcoholism increases vulnerability to AD-related neurodegeneration. Reducing harmful alcohol use may therefore represent a practical approach to lowering long-term dementia risk. This review summarizes the current mechanisms of alcohol induced neuronal damage across different brain regions. Prolonged alcohol consumption accelerates cerebral aging by enhancing oxidative stress, neuroinflammation, disrupting tau protein degradations, and other neuronal damages that intersect with the pathogenesis of AD.

## 1. Introduction

Alzheimer’s disease is a complex neurological disorder, complex and characterized by memory, speech, and mood imbalance. It is a repetitive disruption of specific brain functions and multiple cognitive domains that may ultimately be fatal [1,2]. Neuronal cell death, loss of cholinergic fibers, microglia, and mitochondrial dysfunction, proliferation of reactive astrocytes, repetitive presence of intracellular and extracellular amyloid-β plaques, all have a key role in AD [3,4,5]. These changes take place because of neuronal degeneration, which is related to extracellular neurofibrillary tangles and extracellular senile plaques. The formation of amyloid-β begins with the Amyloid precursor protein (APP), which is found in the neuronal membrane. APP is useful in neuronal growth and repair after injury, and APP needs to be recycled or disposed of like other proteins in the body [6]. In AD, the accumulation of excessive amyloid-β at neuronal synaptic junctions can assemble to form oligomers and subsequently senile plaques, leading to neuronal dysfunction. Tau protein is crucial for stabilizing microtubules; however, its hyperphosphorylation leads to separation from microtubules and the formation of neurofibrillary tangles (NFTs), which can result in microtubule collapse [7,8]. AD also causes dysfunctional cholinergic neurons with a decrease in choline uptake, a decrease in ACh release, and a decrease in choline acetyltransferase (CHAT), which is correlated with the number of plaques and disease severity [9]. With an increased human life span, there are chances of decreasing cognitive functions, which can cause AD-related dementia. AD has become a significant public health concern around the world. Early recognition, inhibition, and therapeutic interventions are required to reduce its distressing effects. The factors associated with higher risk are diabetes, smoking, hypercholesterolemia, hypertension, and chronic alcohol consumption [10]. Among these factors, alcohol-related dementia is very common, and some of the similar cognitive characteristics have also been identified in AD [11,12]. Research indicates that moderate alcohol consumption may confer benefits regarding Alzheimer’s disease; however, persistent alcohol usage can elevate the risk of developing the condition. Memory loss associated with alcohol transpires because of prolonged alcohol misuse. Individuals exhibiting alcohol-related dementia are frequently of advanced age. Like other dementias, it results in a decline of cognitive abilities, brain activity, and irreversible vascular alterations in the brain, characterized by memory impairment [13,14]. Alcohol is a frequently misused substance and a prevalent contributor to neurocognitive impairments, resulting in disruptions of the blood–brain barrier, demyelination of nerve fibers, and synaptic degeneration [15,16,17]. Alcohol excitotoxicity correlates with nutritional depletion, resulting in brain tissue loss and the reduction in cortical-subcortical volume and white matter, hence impairing normal brain function. It mostly involves dopamine transmission within the ventral tegmental area (VTA), nucleus accumbens, and prefrontal cortex, enabling the brain to identify pleasurable acts that warrant recurrence [18].

Chronic alcohol consumption is recognized as a potential risk factor for neurodegeneration; however, not all chronic alcoholics develop AD, and a considerable number of AD patients do not have a history of alcohol misuse, underscoring the multifactorial and heterogeneous nature of AD pathophysiology. Genetic factors markedly affect individual susceptibility, particularly the presence or absence of the APOE ε4 allele, which influences vulnerability to amyloid-β accumulation, tau pathology, and neuroinflammation, leading to diverse outcomes among individuals with similar alcohol exposure [16,17]. The neurological consequences of alcohol depend on dosage, consumption habits, duration, and timing of exposure, with heavy or binge drinking in midlife posing a greater risk than moderate or late-onset consumption. Cognitive decline linked to alcohol intake is frequently affected by vascular and metabolic comorbidities, such as hypertension and cerebral small-vessel disease, which contribute to dementia via mechanisms that may diverge from conventional AD pathology. Furthermore, variations in neuroinflammatory responses, oxidative stress regulation, and neurotrophic support may determine if chronic alcohol-induced damage results in irreversible dementia. The onset of AD in non-alcohol consumers highlights the critical importance of intrinsic disease mechanisms—including aging, genetic predisposition, impaired protein clearance, mitochondrial dysfunction, and chronic low-grade inflammation—indicating that alcohol serves as a modifying rather than a causative factor in the advancement of AD [19,20].

Alcohol modulates both excitatory and inhibitory neurotransmitter pathways. It potentiates inhibitory neurotransmission by positively modulating GABA_A_ receptor function, while simultaneously suppressing excitatory glutamatergic signaling. These effects contribute to downstream activation of the endogenous opioid system, resulting in endorphin release and reinforcement of alcohol reward. The release of β-endorphins following alcohol intake activates μ-opioid receptors, which in turn reduces inhibitory control over dopaminergic neurons in the ventral tegmental area. This disinhibition results in elevated dopamine levels within the nucleus accumbens, strengthening mesolimbic reward signaling and producing the characteristic pleasurable and reinforcing effects of alcohol [21,22,23]. Alcohol also dampens excitatory neurotransmission by interfering with glutamatergic signaling, particularly through inhibition of NMDA receptors [24]. However, the net effect of alcohol on excitatory and inhibitory neurotransmitters is not uniform and depends largely on the brain region and neural circuitry involved. Brain areas such as the nucleus accumbens and the amygdala are especially important in this context, as they are central to reward processing and emotional regulation. Activation of these regions produces feelings of pleasure and reinforcement, which can strengthen alcohol-seeking behavior and increase the risk of relapse [25]. Conversely, in the cerebral cortex, ethanol inhibits cognitive function, resulting in impaired thought processes and diminished voice intelligibility. Alcohol also impedes the behavioral inhibition areas, namely the cerebellum and prefrontal cortex [26,27]. When the prefrontal cortex exhibits decreased activity, individuals experience diminished alertness and increased relaxation. Ethanol also disrupts coordination in the cerebellum, hindering individuals’ ability to walk or execute intricate tasks such as driving [28]. Alcohol influences the hippocampus and pituitary glands, potentially resulting in heightened sexual drive and diminished capacity for sexual engagement [29,30]. The medulla governs autonomic functions like respiration, awareness, and thermoregulation. Alcohol induces drowsiness, depresses respiration, and lowers body temperature to hazardous levels [31]. Alcohol increases cytokine release, activates microglial cells, and elevates inflammatory chemokines, contributing to the formation of amyloid-β plaques and mutations in alpha and beta secretases, resulting in neurodegenerative cell death [32,33]. Alcohol influences the microtubule system, impacting intracellular trafficking by altering MAP2 and hyperphosphorylating tau, which exhibits inclusions in the form of neurofibrillary tangles characteristic of AD [34,35,36]. This review focuses on brain regions commonly affected by both chronic alcohol exposure and AD, explores the links between addictive behaviors and AD, and discusses how long-term alcohol consumption influences the underlying pathophysiology of AD.

## 2. Alcohol-Induced Damage in Key Brain Regions

Alcohol enhances inhibitory neurotransmission by potentiating GABA_A_ receptor activity rather than acting as a direct agonist. This increased GABAergic signaling indirectly engages the endogenous opioid system, promoting the release of endorphins that contribute to the rewarding effects of alcohol. Individuals with alcoholism are more prone to mental problems and behavioral difficulties due to compromised brain functions. The toxicity of alcohol can damage various organs, impairing the functionality of nerve cells in the brain. Research indicates that persistent alcohol consumption affects the anatomical development of the brain [37,38,39]. Moreover, alcohol influences several neurotransmitter systems inside the brain’s reward and stress pathways [40]. These interactions induce immediate reinforcing effects of alcohol and alter neuronal function, leading to the development of alcoholism [39].

### 2.1. Cerebellum

**Figure 1:**
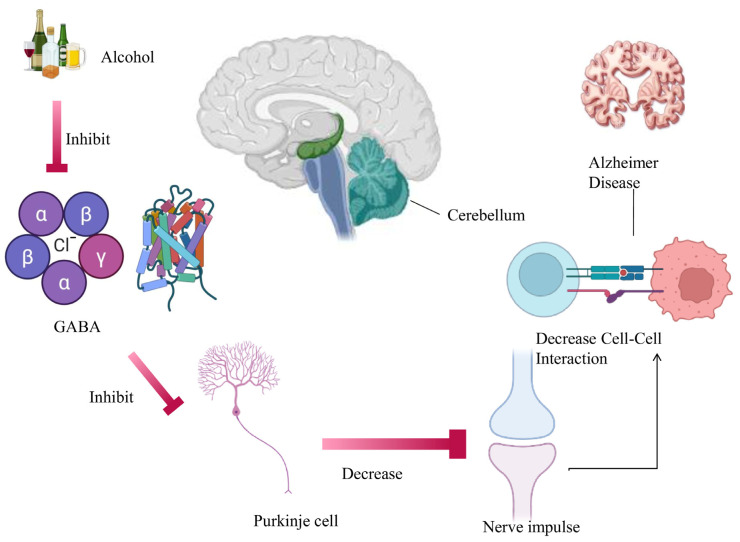
The possible connection between alcohol’s effect on the cerebellum and AD. Excessive alcohol utilization can prompt underlying changes and neuronal damage in the cerebellum, which can increase the risk of cognitive impairment and AD later in life.

Excessive alcohol consumption disrupts hand movements, reaction time, postural stability, balance, and foot-tapping proficiency, with notably detrimental effects on the developing cerebellum. Research indicates that ethanol abstinence results in damage to cerebellar neurons and mitochondria [40,41]. Specialized interneurons within the granule and molecular layers deliver GABAergic inhibitory inputs that modulate the activity of neurons in the cerebellar cortex. Alterations in GABA_A_ receptor-mediated neurotransmission have been associated with the dysfunction of cerebellar activity induced by ethanol [42]. Exposure to ethanol enhances GABA release at Purkinje cell-to-molecular layer interneuron synapses and reciprocal synapses among molecular layer interneurons [43]. Alcohol exerts varying effects on neurotransmitters in the brain, contingent upon their specific locations. The receptors on dopaminergic neurons in the nucleus accumbens bind to these endorphins, resulting in the release of dopamine and serotonin, thereby inducing a euphoric mood. The primary reward centers of alcohol, including the nucleus accumbens and the amygdala, can induce relapse and elicit sensations of bliss. Alcohol suppresses excitatory neurotransmission by interfering with glutamatergic signaling and reducing glutamate receptor activity. As a result, neuronal activity in the cerebral cortex is dampened, leading to impaired cognition, slowed thinking, and difficulties with clear speech. Alcohol also weakens the brain’s inhibitory control systems, particularly within the prefrontal cortex, while disrupting cerebellar function, which together compromise judgment, balance, and the ability to carry out coordinated or complex movements. In addition, chronic alcohol exposure affects the pituitary gland and hippocampus, contributing to altered sexual behavior, including impaired sexual function alongside heightened sexual arousal [44,45,46]. Alcohol induces drowsiness, impaired respiration, and a critically low body temperature in the medulla, which governs autonomic activities such as respiration, thermoregulation, and consciousness [47]. Alcohol stimulates microglial cells, resulting in the production of chemokines and inflammatory cytokines, which contribute to the formation of amyloid-β plaques and mutations in alpha and beta secretases, thus inducing neurodegenerative neuronal death. Alcohol impairs the microtubule system, disrupts intracellular trafficking by modifying MAP2, and induces hyperphosphorylation of tau, all of which contribute to the formation of neurofibrillary tangles (NFTs), a characteristic feature of Alzheimer’s disease (AD). Alcohol induces degenerative alterations in the brain and neuronal death [48,49], Figure 1.

### 2.2. Hippocampus

The hippocampus generates active memory formation signals, whereas the retrosplenial cortex in the occipital lobe aids in long-term object recognition memory. Sharp-wave ripples (SWRs), generated by the CA3 region of the hippocampus, transmit living memory imprints to the neocortex, thereby enhancing memory retention. The retro-splenial brain exhibits the most significant modification of SWR [50,51]. In the “offline” condition of the brain, a greater extent of the cortex is influenced by the excitatory output of SWR, in conjunction with the subcortical nuclei [52]. Dementia is associated with the aggregation of neurofibrillary tangles (NFT) in the hippocampus. The NFTs in the CA2, CA3, and CA4 regions, the stratum lacunosum, and the dentate fasciculus induce synaptic loss. Neocortical prefrontal engram cells are generated during the initial learning phase, utilizing information from the hippocampus, entorhinal cortex, and basolateral amygdala [53]. The maturation of these prefrontal engram cells will ultimately occur with the aid of hippocampal engram cells. Consequently, remote memory retrieval is predominantly dependent on neocortical engram cells [54]. Neurogenesis occurs in the dentate gyrus (DG) of mammals throughout their lifespan, specifically in brain areas that undergo this process. The DG serves as a crucial information conduit in the development of episodic memory inside the hippocampus [48,55]. Alcohol can modify the brain’s functionality and appearance by disrupting its communication networks. Alcohol diminishes the functions of balance, memory, speech, and judgment in the brain, increasing the likelihood of adverse consequences [56,57]. The neurons’ cells diminish in size due to prolonged, excessive consumption. Excessive alcohol consumption can lead to alcohol-induced blackouts. Blackouts are instances of memory loss regarding events that occurred during intoxication. The hippocampus of the brain is momentarily incapable of transferring memories from short-term to long-term storage, a process referred to as memory consolidation. Animal findings support the concept that alcohol impedes memory formation by disrupting neurogenesis and hippocampal function [58]. Chronic drinking negatively impacts the hippocampus by causing neuroinflammation, neuronal death, synaptic dysfunction, and decreased neurogenesis, leading to deficiencies in learning, memory, and cognitive flexibility. Adolescent alcohol consumption may elevate the prevalence of underdeveloped, hyperexcitable synapses in the hippocampus, potentially resulting in memory deficits, excitotoxicity, and other alcohol-related cognitive impairments [59]. Despite the myriad adverse consequences of alcohol on health, alcohol poisoning is particularly detrimental to the brain and neurological system. An essential component in the initiation of neurodegenerative alterations is the hastening of cerebral shrinkage induced by excessive alcohol use [60].

### 2.3. Hypothalamus

**Figure 2:**
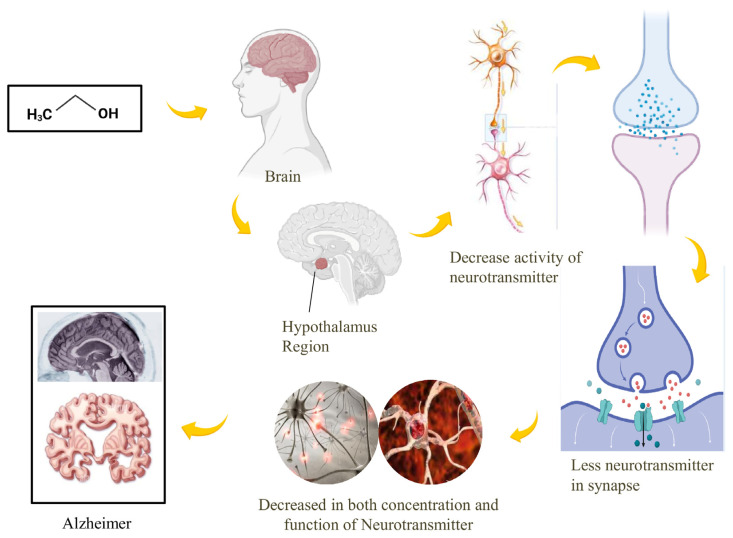
The effect of alcohol on the hypothalamus and its potential role in the development of AD. Alcohol utilization can disturb the ordinary functioning of the hypothalamus, prompting increased oxidative stress, inflammation, and neuronal loss. These effects can contribute to the accumulation of beta-amyloid and tau proteins, which are characteristic of AD.

The hypothalamus is an important part of the brain located below the thalamus, constituting the floor of the third ventricle. That maintains homeostasis by regulating automatic functions, such as body temperature, appetite, thirst, sleep patterns, and emotional responses. It connects the brain and endocrine systems by directing the pituitary gland to release hormones. This regulation includes responses like sweating, shivering, appetite control, stress management, libido, and circadian rhythms, maintaining a stable internal environment by influencing the autonomic nerve system and secreting hormones from numerous glands [61]. Neuromodulator neurochemicals known as neuropeptides play a role in the hypothalamus’s ability to control alcohol consumption [62]. Although they have distinct effects on the drinking response, certain orexigenic neuropeptides promote alcohol consumption in the hypothalamus [63,64]. It appears that these neuropeptides increase alcohol intake in addition to promoting food intake by increasing reward and reinforcement from alcohol [65]. These neuropeptides are raised by alcohol use in a positive response to alcohol, which promotes increased consumption. These stand in contrast to other orexigenic neuropeptides, such as melanin-concentrating hormone and neuropeptide Y, that only infrequently promote alcohol use while occasionally increasing reward. They also differ from neuropeptides that can cause anorexia, such as the endogenous opioid dynorphin, corticotropin-releasing factor, and melanocortin [66]. These neuropeptides work in the hypothalamus to stop people from drinking alcohol and reward them, so they counteract the digestive drive that is promoted by orexigenic neuropeptides. Because of this, even though several hypothalamic neuropeptides cooperate to control different aspects of the alcohol consumption response, Excessive or insufficient signaling from orexigenic or anorexigenic neuropeptides could indeed end up causing consumption of alcohol to become a maladaptive behavior and have a long-lasting impact on the hippocampal region, leading to cognitive impairment [67]. Also, there is evidence that damage to the hippocampal region contributes to cognitive impairment and an increased risk of AD. As a result, hippocampal interventions may be effective in slowing or stopping cognitive loss [68], which is because the cellular injury causes a chronic hyperperfusion state and the accumulation of Amyloid-β, which makes neuronal dysfunction worse and causes cell loss and axonal loss, which leads to AD [69] (Figure 2).

### 2.4. Amygdala

**Figure 3:**
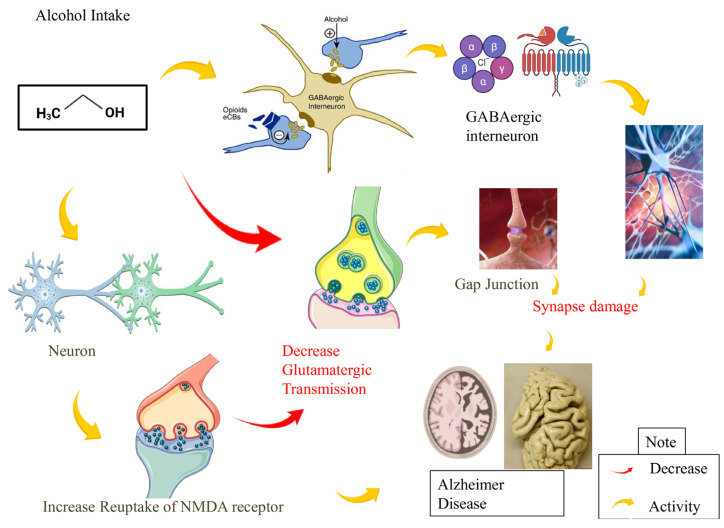
The impact of alcohol on the amygdala and its potential role in the development of Alzheimer’s disease. Alcohol consumption can affect the structure and function of the amygdala, which plays a crucial role in emotional regulation and memory processing. Alcoholism over an extended period can damage GABAergic interneurons, which in turn causes synaptic damage and AD disease. Alcohol also increases NMDA receptor reuptake, which in turn causes AD. Red color represents decrease and the yellow color is shows just the activity happens one after that as a patho-physiological process.

The central nucleus of the amygdala (CeA) may play a key role in modulating alcohol-related behaviors and neuroadaptive mechanisms supplementary to alcohol dependence [70]. Alcohol consumption alters the neurotransmission and amygdala circuitry, leading to changes in emotional processing, anxiety, and stress reactivity. These changes are crucial for the development and maintenance of alcohol dependence. Alcohol supports GABAergic transmission in the CEA through pre- and postsynaptic processes, and constant alcohol helps GABAergic transmission at the pattern levels. Chronic alcohol up-regulates NMDA receptor (NMDAR)-mediated transmission, whereas acute alcohol reduces glutamatergic transmission through actions at N-methyl-D-aspartate (NMDA) and amino-3-hydroxy-5-methyl-4-isoxazolepropionic acid (AMPA) receptors in the CeA [71]. AD patients also exhibit amygdala distortion, significant neuronal loss, and atrophy. The death of neurons, particularly in the magnocellular basolateral amygdala nuclei group, and the repetitive loss of dendrites and axons, trigger amygdaloid atrophy in AD. Through the accumulation of extracellular amyloid peptide deposits, plaques, Lewy bodies, and intraneuronal neurofibrillary tangles, all significantly contribute to atrophy [72]. Although in AD patients, many neurofibrillary tangles and amyloid plaques are found in the accessory basal and cortical nuclei, repetitive in the cortical transition area of the amygdala, although the medial basal nucleus is less damaged [73]. In people with AD, a morphologically distorted structure has been linked to intrinsic damage to the sub-nuclei of the amygdala and their reciprocal connections. Amygdaloid nuclei, which receive information and give birth to hippocampal projections, consistently suffer from neuropathological changes in AD [74,75,76]. Amygdaloid nuclei, however, like the latero-basal nucleus, are less affected because they receive a lot of cholinergic input from the nucleus basalis of Meynert (Figure 3).

### 2.5. Basolateral Amygdala (BLA)

The BLA serves as a convergent hub that integrates emotional sensitivity, reward prediction, and associative memory to inform adaptive behavior, particularly in alcohol-related decision-making. In this context, alcohol use and dependence arise from maladaptive reinforcement of cue–outcome connections encoded in circuits concentrated in the basolateral amygdala, linking the prefrontal cortex, hippocampus, and nucleus accumbens. In aging and AD, the early and gradual susceptibility of the BLA impairs emotional regulation and affective memory, reducing the threshold for stress- and cue-induced alcohol seeking while concurrently undermining cognitive control [77,78,79]. Chronic alcohol exposure mechanistically promotes enduring synaptic remodeling in the BLA, marked by increased glutamatergic activity, compromised GABAergic regulation, dysregulated calcium signaling, and elevated neuronal excitability. These functional alterations are exacerbated by alcohol-induced oxidative stress, microglial activation, and the production of proinflammatory cytokines, which compromise synaptic integrity and diminish plasticity-dependent emotional learning. Significantly, these alcohol-induced molecular and circuit-level disruptions largely coincide with mechanisms associated with Alzheimer’s disease, such as amyloid-β–induced synaptic dysfunction, tau-mediated cytoskeletal destabilization, mitochondrial dysfunction, and the gradual disconnection of limbic–cortical networks [80,81,82,83]. The intersection of alcohol- and Alzheimer’s disease-related pathophysiology within the basolateral amygdala endorses a cohesive mechanistic framework wherein recurrent alcohol consumption hastens age- and disease-associated deterioration of emotional-reward circuits, consequently establishing a feedback loop that intensifies maladaptive drinking behaviors and aggravates neurodegenerative advancement [84].

### 2.6. Hypothalamic–Pituitary–Adrenal (HPA Axis)

The HPA axis is crucial in linking stress response to alcohol use and addiction, with dysfunction indicating a relationship with aging and AD. Alcohol activates the HPA axis, increasing corticotropin-releasing hormone (CRH) and adrenocorticotropic hormone (ACTH) levels, along with cortisol production. Chronic alcohol use leads to maladaptive changes in the HPA axis, characterized by reduced negative feedback and heightened glucocorticoid levels, which alter stress-related brain regions like the amygdala, hippocampus, and prefrontal cortex [59,84]. This results in increased stress sensitivity, reinforcing maladaptive drinking patterns and relapses through enhanced alcohol-related emotional memories and diminished executive function. The HPA axis exhibits pronounced vulnerability during aging and AD, where prolonged glucocorticoid exposure exacerbates synaptic loss in the hippocampus, impairs neurogenesis, and increases amygdala responsiveness. Excessive glucocorticoid signaling at the molecular level leads to oxidative stress, mitochondrial issues, insulin signal impairment, neuroinflammation, and promotes amyloid-β production while reducing its clearance. The disruption of the HPA axis thus creates a vicious cycle, where stress hormones interact with alcohol’s neurotoxic effects, leading to further degeneration in the limbic-cortical network. Ultimately, this analysis reveals the HPA axis as a bidirectional amplifier: alcohol dysregulates stress systems, increasing neurodegeneration risk, while age and AD-related weaknesses in stress regulation foster maladaptive alcohol consumption, reinforcing a harmful feedback loop between addiction and neurodegeneration [78,85].

### 2.7. Prefrontal Cortex (PFC)

PFC is crucial in regulating alcohol consumption and addiction through its control over reward processing, emotional regulation, and decision-making. Its subregions are particularly vulnerable during aging and conditions like Alzheimer’s disease (AD). The PFC integrates signals from limbic areas, such as the amygdala and hippocampus, to manage impulsivity and align behavior with long-term goals. Acute alcohol intake reduces PFC activity, while chronic consumption leads to lasting deficits, including reduced cortical thickness and impaired synaptic functions. This results in diminished inhibitory control and a shift towards compulsive alcohol-seeking behaviors. Chronic alcohol exposure disrupts glutamate balance in the PFC by affecting astrocytic functions and activating NMDA receptors excessively, leading to excitotoxic stress. Additionally, alcohol induces oxidative stress and neuroinflammation, synaptic dysfunction, and PFC communication. These changes correlate with pathological alterations in aging and AD, characterized by synaptic loss and energy metabolism issues, with amyloid-β and tau proteins further impairing PFC functionality and executive functions vital for controlling maladaptive behaviors [60,67,81].

### 2.8. Redox Signaling in AD

Redox signaling functions as a crucial biological link between alcohol consumption and AD, with both conditions marked by a pathological shift from tightly regulated normal redox signaling to sustained oxidative and nitrosative stress that undermines neuronal integrity. In Alzheimer’s disease, mitochondrial dysfunction, compromised antioxidant defenses, and persistent neuroinflammation elevate reactive oxygen and nitrogen species (ROS/RNS), disrupting synaptic plasticity and hastening neurodegeneration; chronic alcohol consumption significantly exacerbates this imbalance by enhancing ROS production via ethanol metabolism pathways, leading to lipid peroxidation, protein oxidation, and DNA damage in already susceptible cortical and hippocampal neurons. Oxidative stress caused by alcohol enhances the amyloidogenic processing of amyloid precursor protein via redox-sensitive activation of β- and γ-secretases, while simultaneously obstructing antioxidant-mediated amyloid clearance, hence accelerating amyloid-β buildup [86]. Furthermore, it promotes tau hyperphosphorylation through the activation of stress-responsive kinases, including GSK-3β and CDK5, alongside the suppression of phosphatase activity, leading to cytoskeletal instability and synaptic loss. At the mitochondrial level, alcohol exacerbates impairments associated with Alzheimer’s disease in electron transport chain functionality, increasing the leakage of reactive oxygen species, reducing ATP synthesis, and disrupting calcium homeostasis, which collectively lowers the threshold for excitotoxic damage. Concurrently, redox imbalance in glial cells triggers microglial activation and astrocytic dysfunction, intensifying proinflammatory signaling and further oxidative damage, while compromising blood–brain barrier integrity and neurovascular redox coupling. Alcoholism in Alzheimer’s disease functions as a redox amplifier, transforming adaptive oxidative signals into persistent oxidative pathology, hence hastening synaptic degradation, network disconnection, and cognitive decline (Table 1) [87].

## 3. Alcohol and Tau Pathology

Alcohol deeply affects the brain, and excessive consumption has long been associated with cerebral damage. Chronic alcohol consumption is associated with irreparable brain damage due to malnutrition, heightened glutamate-induced excitotoxicity, and oxidative stress [75]. The associations between alcohol and these three conditions, as well as their impact on them, differ. Epidemiological studies indicate that moderate alcohol use is associated with a reduced likelihood of developing AD. Minimal to moderate quantities of ethanol protect hippocampal neurons from amyloid toxicity, but excessive ethanol consumption enhances the buildup of amyloid-β and tau phosphorylation. Excessive ethanol consumption accelerates the formation of amyloid-β and tau phosphorylation. Glycogen synthase kinase-3 (GSK3) is associated with amyloid-induced cell death and is essential for the hyperphosphorylation of tau [78]. This complements the regulation of alcohol’s effects. Both short-term and chronic alcohol exposure elevate GSK3 phosphorylation. A recent study revealed that the CA1 sub-region of the hippocampus in a 3xTg-AD mouse brain had hyperphosphorylation at the GSK3 Tau protein site one month after alcohol use. This indicates that frequent alcohol consumption is detrimental to Alzheimer’s disease [79]. Previous research outcomes suggest that moderate to low alcohol intake may safeguard against memory loss (dementia), dementia pathogenesis, and cognitive decline. The principal physiological mechanisms suggested—the antioxidant characteristics of wine flavonoids, their influence on amyloid-β and tau receptors, and alcohol’s protective role against ischemia or stroke—collectively contribute to these potential advantageous effects on the brain. Tau is a constituent of the protein family linked to microtubules and located in neurons [80,81,82]. Alcohol abuse diminishes brain protein breakdown, resulting in pathological alterations and oxidative harm. It also impairs the microtubule system, which is essential for intracellular trafficking [83,84].

**Figure 4:**
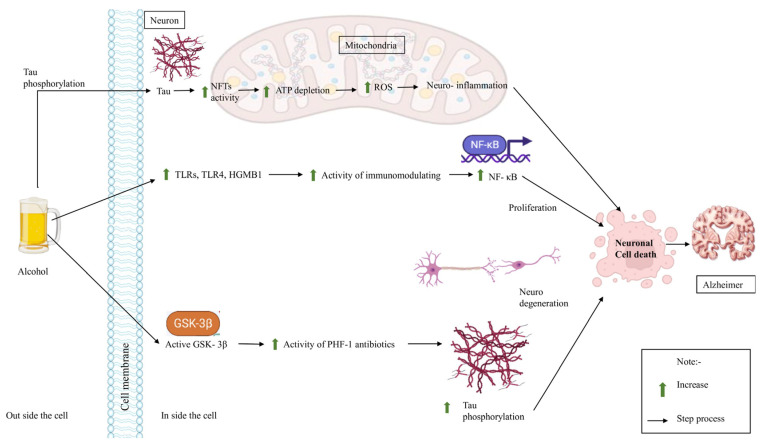
Alcohol’s effect on the Tau receptor in AD. Alcohol increases tau phosphorylation, tau increases NFT activity, which leads to ATP depletion, and increases ROS, causing AD. Alcohol raises TLRS, TLR4, and HGMBI, which raises the activity of immunomodulators and NF-KB, which causes proliferation and AD. Alcohol also makes GSK-3B activate PHF-1, and tau phosphorylation causes neurodegeneration and AD.

Microtubule-associated proteins, including MAP2 and tau, govern the stability of microtubule assembly [85]. Alcohol modifies the phosphorylation of MAP2 and tau proteins. Additionally, alterations in cellular filamentous inclusions of tau are observed in the hepatic histology of alcoholics, while microtubule-associated proteins (MAP2 and tau) govern the stability of microtubule assembly [86]. Alcohol may hinder tau turnover by modifying the activity or expression of proteases and regulators involved in protein breakdown. This may elevate tau levels beyond the permissible range of activity [87]. Another potential association between tau and alcohol is neuroinflammation. Alcohol modifies the phosphorylation of MAP2 and tau. Alterations are observed in cellular filamentous inclusions associated with tau. These benefits are also seen in the alcoholic brain [88]. Alcohol modifies the expression of proteases and regulators of protein degradation, thereby hindering tau turnover and elevating tau levels beyond the acceptable range of activity [5] (Figure 4).

## 4. Neuroinflammation and Microglial Priming

Innate immune cells, or microglial cells, are the brain’s primary immune system and possess surface receptors that identify alien patterns and transmit signals accordingly [89]. This initiates an inflammatory reaction, which is a component of the phagocytic immune response and a factor in healing mediators. Acetaldehyde, a neurotoxic, inflicts irreparable harm on brain structure and function, with some proteins, such as tau, implicated in brain inflammation [89]. Neuroimmune elements in the alcoholic brain include receptors (TLRs), TLR4, various cytokines, and high-mobility group box 1 (HMGB1), which further exacerbate neuroinflammation [90]. It also affects immunity and possesses an immunomodulatory function. The brain supports astrocytes and microglia, which generate neuroimmune factors. Certain studies suggest that acute drinking results in tau accumulation in M1C cells present in humans, correlating with a dose-dependent reduction in cell viability and tau deposition, ultimately causing hippocampus neuronal death. The elevation of NF-κB-mediated transcription of proinflammatory factors is responsible for brain inflammation.

**Figure 5:**
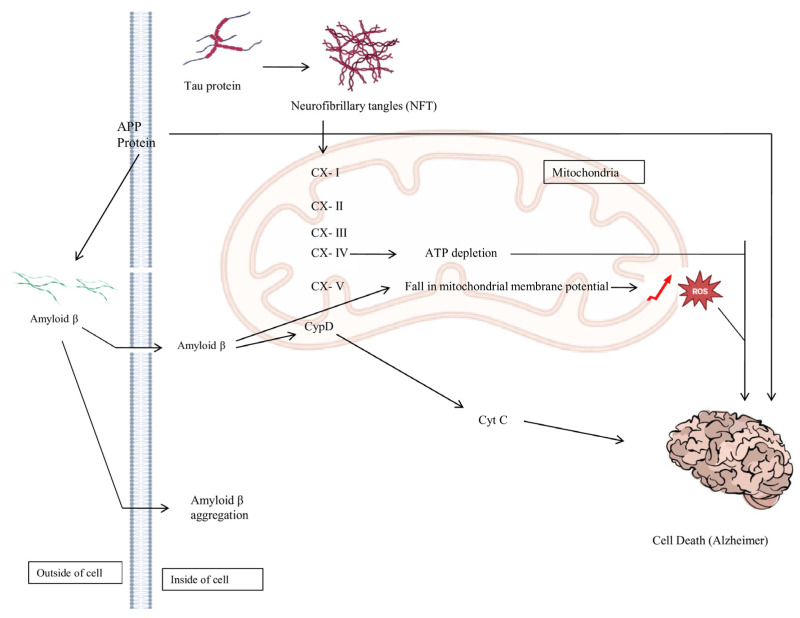
The impact of alcohol on NFT signaling and its potential role in the development of AD. Chronic alcohol consumption can lead to the accumulation of tau protein, which can form NFTs in the brain. NFTs disrupt normal neuronal function and are a hallmark feature of AD. In AD, NFTs spread throughout the brain and contribute to neuronal damage and cognitive impairment. NFTs are thought to disrupt the transport of essential molecules within neurons, leading to cell death and the formation of amyloid plaques.

Alcohol is associated with brain damage due to disrupted synaptogenesis, reduced cellular migration, and compromised cell signaling resulting from defective microtubule formation and tau hyperphosphorylation [91]. Research indicates that the modification of unfolded protein response signaling plays a significant role in the endoplasmic reticulum in Alzheimer’s disease neurodegeneration, while oxidative damage is another contributor to cerebral impairment resulting from alcohol’s neurotoxicity [92,93,94]. Alcohol consumption causes neuronal apoptosis and endoplasmic reticulum (ER) stress. Alcohol consumption mitigates endoplasmic reticulum stress and neuronal apoptosis via inhibiting ER stress. A study demonstrated tau phosphorylation and cleavage in postnatal day 7 (P7) mice, concluding that acute alcohol exposure elevated tau phosphorylation, or hyperphosphorylation, as seen by antibodies PHF-1 and PHF-1a, which correspond to GSK-3 phosphorylation epitopes. While ethanol increases tau phosphorylation through GSK-3 activation, the potential involvement of additional protein kinases or phosphatases cannot be disregarded [95,96,97]. The control of tau phosphorylation and dephosphorylation may vary based on the specific phosphorylation site. In the rat brain, GSK-3 is predominantly expressed in neurons, especially in neurites until postnatal day 21, indicating that ethanol-activated GSK-3 may phosphorylate tau in neurites (Figure 5) [98,99,100].

## 5. Oxidative Stress and Mitochondrial Dysfunction

**Figure 6:**
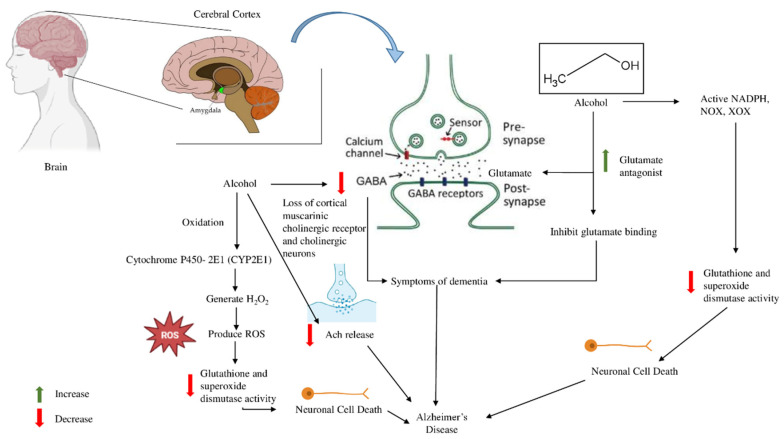
Alcohol oxidation results in the production of cytochrome (P450-CYP2E1) and reactive oxygen species (ROS), which can result in decreased glutathione and the activity of superoxide dismutase, causing neuronal cell death and AD. Alcohol reduces the loss of cortical muscarinic cholinergic receptors and increases the glutamate antagonist activity of NOX, NADPH, and XOX, resulting in neuronal cell death and AD.

The impact of ethanol on brain oxidative stress has established a connection to the pathophysiology of neurodegenerative disorders such as Alzheimer’s disease. This connection among oxidative stress, endoplasmic reticulum stress, and autophagy is what occurs. Oxidation of ethanol by cytochrome P450-2E1 (CYP2E1) generates H2O2, which can combine with copper or iron to form reactive oxygen species (ROS) [101,102]. Human neurons exhibit a twofold elevation in CYP2E1 activity and a concomitant rise in ROS generation with exposure to 17.5 mM ethanol [103,104]. Prolonged alcohol consumption has been associated with elevated CYP2E1 expression in humans, rats, and the cerebral cortex, accompanied by diminished glutathione and superoxide dismutase function [66]. Acetaldehyde, a byproduct of ethanol produced by alcohol dehydrogenase, can activate NADPH oxidase (NOX) and xanthine oxidase (XOX), which are primary sources of superoxide generation and significant contributors to oxidative stress. Animal studies indicated that rat synaptosome membranes had elevated amounts of protein carbonyls and malondialdehyde (MDA) following alcohol treatment. Research indicates that glutathione levels, along with synaptosomal activity of catalase and superoxide dismutase, decline in rats administered 20% (*v*/*v*) ethanol at a dosage of 5 g/kg body weight daily for sixty days. The ensuing oxidative stress alters lipid composition and compromises the functionality of synaptosomal membranes. Redox signaling is essential in the central nervous system, governing mitochondrial metabolism and synaptic plasticity. However, aging disrupts redox homeostasis, leading to oxidative stress, which increases neuronal vulnerability. In Alzheimer’s disease (AD), oxidative stress contributes to neurodegeneration, exacerbating amyloid pathology and tau hyperphosphorylation. Alcohol consumption further disrupts redox balance, enhancing neuroinflammation and cognitive decline by depleting antioxidant defenses and promoting oxidative damage. This interaction forms a dual-hit model where aging and AD create a primary vulnerability, and alcohol acts as an additional stressor, accelerating neurodegeneration [105,106] (Figure 6).

## 6. Cholinergic Dysfunction

The cholinergic system is an essential neurotransmitter system in animal brains. Neurotransmitters, including acetylcholine (Ach), are essential for memory and learning. Ethanol has the potential to harm cholinergic neurons in the brain. Alcohol misuse can lead to the destruction of cortical muscarinic cholinergic receptors and cholinergic neurons; mild alcohol intake in rats (0.8 g/kg) enhances ACh release in the hippocampus, while high alcohol intake (2.4 g/kg) produces the contrary effect [107]. This indicates that the suppression of acetylcholine release by ethanol may be the underlying factor in cognitive impairment associated with alcohol consumption. Similarly, the release of acetylcholine and the activity of choline acetyltransferase diminish in the cerebral cortex, hippocampus, and cerebellum of adult rats that have been given ethanol (20% *v*/*v*) as their exclusive drinking water for a duration of three to six weeks [108]. Studies indicate that high quantities of ethanol can inhibit ACh release by diminishing its production, perhaps worsening dementia symptoms in Alzheimer’s disease patients. However, ethanol at a low dose can enhance ACh release, perhaps safeguarding the nervous system and mitigating AD symptoms [109].

## 7. Amyloid-β Metabolism and Clearance

**Figure 7:**
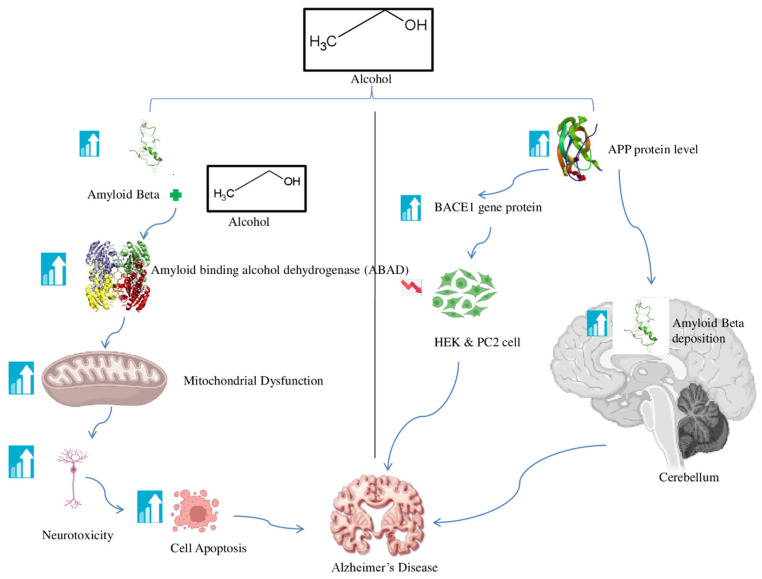
The relationship between alcohol, amyloid-β, and Alzheimer’s disease. Excessive alcohol consumption can lead to the accumulation of amyloid-β, a toxic protein that forms clumps or plaques in the brain. Amyloid-β aggregation causes mitochondrial dysfunction, neurotoxicity, and cell apoptosis, and an increase in the APP and BAC1 gene protein in HEK and PC2 cells is other factors that contribute to AD.

Epidemiological studies indicate that excessive alcohol intake leads to the formation of amyloid-β and tau phosphorylation, while moderate alcohol consumption safeguards hippocampal neurons against amyloid-β toxicity [110,111]. Amyloid-β significantly contributes to Alzheimer’s disease, as mitochondrial dysfunction occurs in the first stage of the condition [112]. Mitochondria are crucial for free radical production, maintaining cellular homeostasis, and modulating energy metabolism. The neurotoxicity induced by the protein results from the interaction between amyloid-binding alcohol dehydrogenase (ABAD), which modulates amyloid-β binding and activates the signaling cascade that culminates in neuronal death [113]. ABAD is claimed to play a crucial function in regulating estrone levels in neurons, hence stimulating apoptotic levels [114]. Moderate alcohol intake may diminish amyloid-β synthesis, but substantial alcohol use is associated with Alzheimer’s disease due to the aldehyde mechanism within the amyloid cascade theory, exacerbated by APP overproduction in transgenic mice [115]. The levels of APP protein were compared between alcohol-fed APP processing and chronic alcoholism, with effects shown in several regions of the rat brain [116]. APP is a membrane protein characterized by three distinct types of cleavage secretases. This breakage results in the development of amyloid plaques in the central nervous system. APP has been shown to enhance its activity, resulting in amyloid-β deposition, while acute or moderate drunkenness may impede amyloid-β deposition [117,118]. Gene expression may also facilitate the formation of amyloid-β [119,120]. APP protein levels were significantly elevated in the cerebellum, hippocampus, and striatum of the alcohol-fed group relative to the control group. The group subjected to persistent alcohol use had increased levels of APP protein in both the frontal and cerebral cortices. These investigations indicate that prolonged ethanol use can disrupt the expression levels of APP and its processing enzymes, as senile plaques in the brain lead to the sequential cleavage of APP by beta-secretase (BACE1) and gamma-secretase. Amyloid-β is pivotal in cerebral function. Amyloid-β can aggregate into oligomers that may harm neurons and synapses. In vitro studies indicate that binge drinking elevates APP and BACE1 protein levels in the cerebellum and hippocampus, while reducing the toxicity of HEK and PC2 cell lines [121,122] Figure 7.

## 8. Conclusions and Public Health Implications

The hippocampus, amygdala, and prefrontal cortex are key brain regions involved in AD pathology and are also particularly vulnerable to the effects of alcohol. Damage to these regions can give rise to a wide range of cognitive and behavioral disturbances, which over time may increase the risk of developing AD. While some studies have suggested that moderate alcohol intake could exert limited protective effects on brain health, the available evidence remains inconsistent. In contrast, excessive or long-term alcohol consumption is clearly associated with detrimental changes in brain structure and function, with important implications for AD progression. Despite increasing interest in this area, the relationship between alcohol use and AD is still not fully understood. This highlights the need for further investigation into the mechanisms by which repeated alcohol exposure affects susceptible brain regions and accelerates disease-related pathology. Future research should carefully consider factors such as the duration, frequency, and pattern of alcohol consumption, as well as individual variability in vulnerability to alcohol-induced brain damage. From a broader perspective, this review emphasizes the importance of limiting alcohol intake and maintaining a healthy lifestyle as potential strategies to support brain health and reduce the risk of AD.

Although substantial progress has been made in understanding alcohol-induced neurotoxicity in the context of AD, several critical questions remain unresolved. In particular, the interaction between genetic risk factors—most notably the APOE4 allele—and chronic alcohol exposure is poorly defined. How APOE4 modifies alcohol-related tau hyperphosphorylation, amyloid-β accumulation, synaptic dysfunction, and neuroinflammatory responses warrants focused investigation. Addressing these questions through well-designed preclinical studies and human cohort analyses may provide valuable mechanistic insight and help identify novel targets for intervention aimed at mitigating alcohol-associated worsening of AD. Continued research in this area has the potential to advance our understanding of AD pathophysiology and inform future prevention and treatment strategies.

## Figures and Tables

**Table 1:** Summary of Preclinical Evidence Linking AD Models, Alcohol Exposure, and Redox Signaling.

AD Model	Alcohol Exposure Protocol	Main Outcomes (Behavior, Pathology, Inflammation, Redox)	Major Conclusions	References
APP/PS1 mice	Chronic ethanol in drinking water (4–6 months)	Worsened spatial memory, increased Aβ burden, elevated lipid peroxidation, and reduced antioxidant enzymes.	Alcohol accelerates amyloid pathology and oxidative stress in genetically vulnerable brains.	[84,85]
3xTg-AD mice	Intermittent or chronic ethanol exposure	Enhanced tau phosphorylation, synaptic loss, increased microglial activation, and mitochondrial dysfunction	Alcohol amplifies both Aβ and tau pathology via redox-inflammatory mechanisms.	[84,85]
Tau transgenic models	Long-term ethanol administration	Increased tau aggregation, oxidative protein damage, impaired motor and cognitive function	Redox imbalance links alcohol exposure to tau-driven neurodegeneration	[84,85]
Aged wild-type mice	Chronic or binge-like ethanol exposure	Cognitive deficits, neuroinflammation, glutathione depletion, mitochondrial ROS elevation	Aging reduces redox reserve, increasing sensitivity to alcohol-induced brain damage.	[84,85]
AD + aging models	Alcohol exposure during mid- to late-life	Exaggerated synaptic loss, impaired neurovascular coupling, and BBB disruption	Alcohol acts as a second hit that accelerates age- and AD-related redox failure.	[84,85]

## Data Availability

No new data were created or analyzed in this study. Data sharing does not apply to this article.

## Abbreviations

ABAD	Amyloid β Binding Alcohol Dehydrogenase
Aβ	Amyloid-β
ACh	Acetylcholine
AD	Alzheimer’s Disease
AMPA	α-Amino-3-Hydroxy-5-Methyl-4-Isoxazolepropionic Acid
APP	Amyloid Precursor Protein
ATP	Adenosine Triphosphate
BACE-1	Beta-Site APP-Cleaving Enzyme-1
CA1/CA2/CA3/CA4	Cornu Ammonis Regions of the Hippocampus
CeA	Central Nucleus of the Amygdala
ChAT	Choline Acetyltransferase
CNS	Central Nervous System
CRF	Corticotropin-Releasing Factor
CYP2E1	Cytochrome P450 2E1
DG	Dentate Gyrus
DNA	Deoxyribonucleic Acid
ER	Endoplasmic Reticulum
GABA	Gamma-Aminobutyric Acid
GABA_A_	Gamma-Aminobutyric Acid Type A Receptor
GSK-3/GSK-3β	Glycogen Synthase Kinase-3 Beta
HEK	Human Embryonic Kidney
HMGB1	High-Mobility Group Box-1
H_2_O_2_	Hydrogen Peroxide
MAP2	Microtubule-Associated Protein 2
MCH	Melanin-Concentrating Hormone
MDA	Malondialdehyde
mPFC	Medial Prefrontal Cortex
mTOR	Mammalian Target of Rapamycin
NMDA/NMDAR	N-Methyl-D-Aspartate (Receptor)
NFTs	Neurofibrillary Tangles
NF-κB	Nuclear Factor Kappa-Light-Chain-Enhancer of Activated B Cells
NOX	NADPH Oxidase
NPY	Neuropeptide Y
PFC	Prefrontal Cortex
PHF-1	Paired Helical Filament-1
POMC	Pro-opiomelanocortin
RNA	Ribonucleic Acid
ROS	Reactive Oxygen Species
SWRs	Sharp-Wave Ripples
TLR/TLR4	Toll-Like Receptor/Toll-Like Receptor 4
XOX	Xanthine Oxidase
